# Changes in characteristics and HIV‐clinical outcomes of pregnant people living with HIV in the UK

**DOI:** 10.1111/hiv.70056

**Published:** 2025-06-10

**Authors:** H. Okhai, A. Baskaran, L. Bukasa, H. Peters, C. Thorne, C. Sabin, the UK Collaborative HIV Cohort

**Affiliations:** ^1^ Institute for Global Health University College London London UK; ^2^ National Institute for Health and Care Research (NIHR) Health Protection Research Unit (HPRU) in Blood‐borne and Sexually Transmitted Infections at University College London London UK; ^3^ Great Ormond Street Institute of Child Health University College London London UK

**Keywords:** HIV, outcomes, postpartum, pregnancy

## Abstract

**Objective:**

In this study, we explore changes in the demographic and clinical characteristics of pregnant people living with HIV, and their post‐partum HIV outcomes between 2000 and 2018.

**Methods:**

We described pregnancies resulting in a live birth from the linked UK CHIC/ISOSS dataset in three calendar periods (2000–2006; 2007–2012; 2013–2018). Thereafter, we explored median CD4 count change and viral rebound from delivery to 12 months post‐partum.

**Results:**

In total, 4341 pregnancies were included. Maternal age increased from 31 (IQR: 28–35) years in 2000–2006 to 34 (IQR: 30–37) years in 2013–2018. Those conceiving in the most recent period had been diagnosed with HIV for longer (2000–2006: 3.0 years to 2013–2018: 7.5 years), had a higher median CD4 count (431–583 cells/mm^3^), and median nadir CD4 count (219–260 cells/mm^3^); they were also more likely to have initiated ART prior to estimated conception (70.1%–92.3%), and have a suppressed conception viral loads (VL) (56.6%–82.0%).

There was no difference in median CD4 count change over the three calendar periods (2000–2006: +60 [IQR: −44, +179]; 2007–2012: +51 [−45, +172]; 2013–2018: +28 [−100, +175] cells/mm^3^; *p* = 0.12). The cumulative proportion of people with viral rebound at 12 months post‐delivery was reduced in 2013–2018 (8.2%) when compared with previous periods (2000–2006: 28.1%; 2007–2012: 19.5%).

**Conclusion:**

Clinical management of pregnant people has changed over time, resulting in positive trends in this study both within pregnancy and post‐partum. Further work needs to understand what barriers remain for those who do not achieve optimal management of HIV in the post‐partum period.

## INTRODUCTION

In the UK, the universal recommendation of antenatal HIV screening, high uptake of effective antiretroviral therapy (ART) and enhanced clinical care pathway (during pregnancy, at birth and in the post‐partum period) has reduced the rate of vertical transmission of HIV to below 0.3% [1]. As a result, there has been an increase in the number of subsequent pregnancies amongst people known to be living with HIV. Annually, there were 1000–1400 pregnancies reported in people living with HIV across the UK between 2006 and 2013 [2], with around 800–900 pregnancies in the UK in 2014–2018. The Integrated Screening Outcomes Surveillance Service (ISOSS), previously the National Surveillance of HIV in Pregnancy and Childhood (NSHPC), has been collecting population‐level data for more than 30 years on pregnancies in people diagnosed with HIV by delivery [3]. By linking this surveillance data to a large clinical cohort of people accessing HIV care services in the UK, we explore changes in the demographic and clinical characteristics at the estimated time of conception of pregnant people living with HIV and their post‐partum HIV clinical outcomes between 2000 and 2018.

## METHODS

The UK Collaborative HIV Cohort (UK CHIC) Study was a cohort of individuals with diagnosed HIV (aged >16 years) who accessed care at one or more of 25 HIV clinics in the UK at any time between 1996 and 2018. At close of study, approximately one‐third of all people seen for HIV care in the UK were included in the cohort. Study methods are described elsewhere [4]. In brief, clinics collected data on an annual basis on demographic information, ART history, laboratory results, and AIDS diagnoses amongst all people attending their clinic for HIV care. The UK CHIC Study has ethics approval (MREC/00/7/47).

Comprehensive population‐based active surveillance is available on all pregnancies amongst people with HIV diagnosed in the UK since 1989. This was carried out initially by the NSHPC from 1989 to 2018, and subsequently by ISOSS, commissioned by NHS England as part of the NHS England Infectious Diseases in Pregnancy Screening Programme [3]. Reports of all pregnancies in women living with HIV diagnosed by the time of delivery are submitted to ISOSS by NHS maternity and paediatric respondents at each unit nationally via a secure online portal; these reports include data on sociodemographics, treatment, CD4+ T‐cell counts (‘CD4 count’) and HIV viral loads (VL) in pregnancy, as well as on delivery and outcomes. Data are collected under legal permissions granted under Regulation 3 of the Health Service (Control of Patient Information) Regulations 2002.

Linkage between the two datasets was undertaken annually using a deterministic algorithm that utilized basic demographic and clinical data [5]. The analyses described here are based on the final UK CHIC dataset (data collected up to 31 December 2018) where 21.2% (5080/24017) of pregnancies in the ISOSS dataset were successfully linked to the UK CHIC Study database. We considered all full‐term (≥37 weeks) pregnancies resulting in a live birth reported between 2000 and 2018 amongst those diagnosed with HIV prior to their pregnancy. Estimated conception date was calculated using the estimated delivery date (estimated delivery date *minus* 266 days).

Demographic (age at estimated conception, ethnicity [White, Black Any, Asian Any, Other/Unknown]), HIV‐clinical factors at estimated conception (time since HIV‐diagnosis, time since ART initiation, CD4 count, nadir CD4 count, undetectable viral load [VL≤50 copies/mL], prior diagnosis of Hepatitis B/C virus at estimated conception) and pregnancy‐related (birth outcome [live‐single, live‐twins], parity at conception [0, 1, 2, ≥3]) factors were described over the study period.

We assessed two HIV‐clinical outcomes in the 12‐month post‐partum period:

- *Change in CD4 count:* assessed amongst pregnant people without a subsequent pregnancy recorded in the 12 months post‐delivery. Those included were required to have a CD4 count measure available prior to the estimated conception date (calculated as 266 days before estimated delivery date), and 12 months post‐delivery (last available CD4 count between 9 and 15 months post‐delivery).
- *VL rebound*: assessed amongst pregnant people who had initiated ART and had an undetectable VL before delivery. VL rebound was defined as the first of two consecutive HIV VL measures >50 copies/mL within 12 months post‐delivery.

Baseline characteristics and HIV clinical outcomes were assessed across three time periods: 2000–2006, 2007–2012 and 2013–2018. Changes in factors over time were assessed using linear (continuous outcomes) or logistic (categorical outcomes) regression. The change in CD4 count was described using the median and interquartile range (IQR) in each time period, with differences tested for significance using a Kruskal–Wallis test. We estimated the proportion of women with VL rebound within 3, 6 and 12 months post‐delivery using the Kaplan–Meier method, with follow‐up time additionally right‐censored at the earliest of the following: estimated conception date if a subsequent pregnancy was recorded, 12 months post‐delivery, or the last date of clinical follow‐up/UK CHIC administrative censoring date. Patterns of VL rebounds in each time period were compared using the log‐rank test.

## RESULTS

### Study sample

In total, 4341 full‐term pregnancies from the ISOSS database were successfully linked with UK CHIC and were used in this analysis. Pregnancies included were from 3073 people living with HIV, with 1095 (25.2%), 1923 (44.3%) and 1323 (30.5%) pregnancies reported in 2000–2006, 2007–2012 and 2013–2018, respectively (Table 1).

**TABLE 1:** Demographic, HIV‐clinical and pregnancy‐related characteristics of pregnant people living with HIV, stratified by time period of estimated conception.

		All pregnancies	Time period of estimated date of conception			
			2000–2006	2007–2012	2013–2018	*p*‐value^c^
*N*		4341	1095	1923	1323	
**Demographic factors**						
Age at estimated conception (years), median (IQR)		32 (28–36)	31 (28–35)	32 (28–36)	34 (30–37)	<0.0001
Ethnicity, *n* (%)	White	558 (12.9%)	146 (13.3%)	239 (12.4%)	173 (13.1%)	0.74
	Black Any	3517 (81.0%)	888 (81.1%)	1591 (82.7%)	1038 (78.5%)	0.01
	Asian Any	66 (1.5%)	13 (1.2%)	32 (1.7%)	21 (1.6%)	0.58
	Other/Unknown	200 (4.6%)	48 (4.4%)	61 (3.2%)	91 (6.9%)	<0.0001
**HIV‐clinical factors**						
Time since HIV diagnosis (years), median (IQR)^a^		5.0 (2.5–8.3)	3.0 (1.6–5.5)	5.0 (2.7–7.5)	7.5 (4.2–10.7)	<0.0001
ART initiation before estimated conception, *n* (%)		3630 (83.6%)	775 (70.8%)	1629 (84.7%)	1226 (92.7%)	<0.0001
Time since ART initiation (years), median (IQR)^b^		4.1 (2.1–6.8)	2.7 (1.4–4.6)	3.9 (2.0–6.5)	5.5 (3.0–8.9)	<0.0001
Undetectable viral load, *n* (%)^a^		2580 (64.1%)	439 (46.1%)	1136 (63.2%)	1005 (79.1%)	<0.0001
CD4 count prior to estimated conception (cells/mm^3^), median (IQR)^a^		503 (360–670)	431 (290–590)	490 (361–653)	583 (430–755)	<0.0001
Nadir CD4 count prior to estimated conception (cells/mm^3^) median (IQR)^a^		235 (130–358)	219 (110–350)	228 (130–346)	260 (140–383)	<0.0001
AIDS diagnosis prior to estimated conception, *n* (%)		711 (16.4%)	212 (19.4%)	310 (16.1%)	189 (14.3%)	0.003
Diagnosis of Hepatitis B or C prior to estimated conception, *n* (%)		156 (3.6%)	37 (3.4%)	74 (3.8%)	45 (3.4%)	0.72
**Pregnancy‐related factors**						
Birth outcome, *n* (%)	Single birth	4228 (97.4%)	1063 (97.1%)	1882 (97.9%)	1283 (97.0%)	0.22
	Twins	113 (2.6%)	32 (2.9%)	41 (2.1%)	40 (3.0%)	
Parity, *n* (%)^a^	0	846 (20.8%)	241 (24.2%)	360 (20.0%)	245 (19.2%)	0.0008
	1	1535 (37.7%)	386 (38.8%)	687 (38.1%)	462 (36.2%)	0.39
	2	1043 (25.5%)	225 (22.6%)	477 (26.5%)	341 (26.7%)	0.05
	≥3	653 (16.0%)	144 (14.5%)	279 (15.5%)	230 (18.0%)	0.05

*Note*: Based on pregnancies, therefore a woman can be represented more than once. %: percentage.

^a^ Missing: Undetectable viral load‐319, CD4 count‐392, Nadir CD4 count‐270, Time since HIV diagnosis‐74, Parity‐264.

^b^ Note: Based on ART initiation pre‐pregnancy (*n* = 3605).

^c^ Based on linear or logistic regression as required.

The median age at conception was 32 years (IQR: 28–36 years). Most pregnancies were amongst people from a Black ethnic background (81.0%), and women who had been diagnosed with HIV for a median of 5.0 years (IQR: 2.5–8.3) at conception. Median CD4 count and nadir CD4 count at the time of conception were 503 cells/mm^3^ (IQR: 360–670 cells/mm^3^) and 235 cells/mm^3^ (IQR: 130–358 cells/mm^3^), respectively. For 83.6% of pregnancies, ART had been started before conception, with conception VL undetectable in 69.0%. For most people, the pregnancy resulted in a single birth (97.4%).

### Changes in the characteristics of women at conception

Maternal age increased from 31 (IQR: 28–35) years in 2000–2006 to 34 (IQR: 30–37) years in 2013–2018 (*p* < 0.0001). Although there were some slight changes in the ethnicity distribution of women over time, this was largely explained by a slight increase in the proportion of those who were of other/unknown ethnicity in the later time period (2000–2006: 4.4%; 2013–2018: 6.9%; *p* < 0.0001). Women conceiving in the most recent period had been diagnosed with HIV for longer (2000–2006: 3.0 years; 2013–2018: 7.5 years; *p* < 0.0001). Both the median CD4 count and median nadir CD4 count at conception increased over the period (CD4‐count: from 431 to 583 cells/mm^3^, *p* < 0.0001; nadir CD4: from 219 to 260 cells/mm^3^, *p* < 0.0001). There were also increases in the proportion of pregnancies conceived on ART (from 70.8% to 92.7%, *p* < 0.0001) and, of those, with suppressed VL at conception (from 57.0% to 80.5%, *p* < 0.0001), with a reduction in the proportion of those with an AIDS diagnosis prior to conception (from 19.4% to 14.3%, *p* = 0.003).

### HIV‐clinical outcomes

CD4 counts at both conception and 12 months post‐partum were available for 1674 of the 4341 (38.6%) pregnancies. Amongst this group, there was a median change in CD4 count of +52 [−50, +175] cells/mm^3^, with no significant difference in this change over the three calendar periods (Table 2; 2000–2006: +60 cells/mm^3^ [IQR: −44, +179 cells/mm^3^]; 2007–2012: +51 cells/mm^3^ [IQR: −45, +172 cells/mm^3^]; 2013–2018: +28 cells/mm^3^ [IQR: −100, +175 cells/mm^3^]; *p* = 0.12).

**TABLE 2:** Median change in CD4 count and cumulative proportion with viral rebound within 12 months post‐partum, stratified by year of estimated date of conception.

Year of estimated conception	Median change in CD4 count, median (IQR)	Cumulative proportion with viral rebound (%)
*N*	1674	2857
2000–2006	+60 (−44, +179)	28.1%
2007–2012	+51 (−45, +172)	19.5%
2013–2018	+28 (−100, +175)	8.2%
*p*‐value	0.12	<0.0001

Approximately two‐thirds (65.8%; 2857/4341) of women had complete VL data and were, therefore, included in the VL rebound analysis. Of this group, 10.5%, 14.4% and 18.0% experienced a VL rebound by three, six and 12 months post‐delivery, respectively. The cumulative proportions of those experiencing viral rebound at three, six and 12 months post‐delivery were reduced in the most recent time period (Table 2; 2013–2018: 3 months 4.6%, 6 months 6.6% 12 months 8.2%) when compared with 2000–2006 (3 months 17.0%, 6 months 22.8% 12 months 28.1%).

## DISCUSSION

Using data from one of the largest observational cohorts linked to a national surveillance programme, we have described the changing characteristics of women living with HIV who gave birth between 2000 and 2018. We report key changes around people's HIV‐related clinical status. Although we found no evidence of a change in CD4 count (when compared with pre‐pregnancy CD4 count) 12 months after delivery, in recent times there has been a decrease in the rate of viral rebound in the first year post‐partum.

Like many other settings, we found a greater number of repeat pregnancies in later years, coincident with changes in attitudes and intention towards pregnancy amongst people with HIV [6, 7]. In our study population, most were from a Black ethnic minority. In the general population, it has been well documented that there are continued inequalities in maternal and infant outcomes amongst those from Black and Minority ethnic populations which may be driven by barriers to accessing antenatal care [8]. Even in the context of the UK where antenatal care is provided free of charge by the NHS, language barriers, lack of awareness or poor understanding of how to access services, and lower economic status have been shown to impede access to antenatal care [9]. These factors must be considered when caring for pregnant people of diverse ethnic backgrounds living with HIV as experiences with healthcare services during pregnancy may determine how well they engage with their HIV care into the post‐partum period.

Given this population is ageing, the large increase in time since HIV diagnosis across the three time periods was expected. This is a testament to the excellent quality of pre‐pregnancy care as we see an increase in the proportion of women conceiving on ART who also had an undetectable viral load. Previous analyses of the linked UK CHIC and historic NSHPC cohort have demonstrated there may be positive effects of care during pregnancy on engagement in HIV care in the post‐partum period [10]. Promoting health knowledge has been shown to empower women [11]; this may suggest that positive experiences with clinical teams could have inspired people with HIV to make confident healthcare‐related decisions regarding their pregnancy.

The post‐partum period can be a challenging time in general with increased physiological changes, risk of post‐partum depression and new/realigned responsibilities. For people with HIV, this can also mean an increased risk of suboptimal ART adherence [12, 13], which can result in immunosuppression. We found no difference in CD4 count in the post‐partum period when compared with pre‐pregnancy. However, this must be interpreted with caution, as this is based on a small proportion of pregnancies that were successfully linked between the two databases.

Previous data from this cohort has shown pregnant people receiving ART whose VLs are suppressed are at increased risk of viral rebound when compared with a control population [14]. Here, we have demonstrated a reduction in the rate of viral rebound in the post‐partum period, particularly in the most recent time period. Although this is a success, it is important to acknowledge that the proportion of those with a VL rebound is not negligible and remains higher when compared with all people with HIV [15, 16]. Further research into the risk factors associated with viral rebound amongst this vulnerable group must be explored to improve provision of adherence support and care delivery.

Though a key strength of this analysis is the use of linked datasets describing women with HIV who have been pregnant in the UK, we must highlight the limitations of this study. Although the UK CHIC study provides a representative sample of people living with HIV, if a woman moved to another centre outside of the collaboration, there would be no further record; therefore, any subsequent pregnancies would not be included in this analysis. UK CHIC/ISOSS datasets are linked using a deterministic/data‐driven approach based on the data at the time [5]; therefore, if data are missing or incorrectly submitted to either cohort, this may result in undermatching. We did not include pre‐term births. Women who experience a pre‐term birth undergo further stressors which are likely to impact their HIV‐related outcomes. For this study, we felt it was important to only consider full‐term births to ensure we did not inadvertently introduce a key confounder in our results. Finally, ISOSS was linked to the last available UK CHIC dataset in 2018. Although this is the only dataset that can comment on the long‐term outcomes of women in the post‐partum period in this setting, unfortunately, we are unable to comment on the rate of VL rebound with the most recent treatment regimens.

As the characteristics of women conceiving with HIV change, healthcare professionals responsible for their care must be aware of the increased risk of viral rebound post‐partum to ensure that all pregnant people with HIV have the best possible post‐partum outcomes. Although the rate of VL rebound in the post‐partum period has decreased dramatically in the most recent years of this analysis, further work is required to understand how we can help pregnant people with HIV remain adherent to treatment and reduce the risk of rebound.

## AUTHOR CONTRIBUTIONS

HO was responsible for conceptualisation, formal analysis, investigation, methodology, visualization, writing of the original draft, and reviewing and editing with support from AB. HP and CT were responsible for validation, and writing, reviewing, and editing. TH was responsible for data curation, and writing, reviewing, and editing. CAS was responsible for conceptualisation, funding acquisition, supervision, methodology, validation, and writing, reviewing, and editing. HO, CAS, and HP had full access to all the data in the study, and HP and CAS verified the data. All authors had final responsibility for the decision to submit for publication.

## FUNDING INFORMATION

This work was supported by the Medical Research Council, UK (Grant nos: G0000199, G0600337, G0900274 and M004236). This research was supported by the National Institute for Health and Care Research Health Protection Research Unit (NIHR HPRU) in Blood Borne and Sexually Transmitted Infections at University College London in partnership with the UK Health Security Agency (HPRU Grant no: NIHR200911). This work is partly funded by the NIHR GOSH BRC. The views expressed are those of the authors and not necessarily those of the National Institute for Health Research, the Department of Health and Social Care, or UK Health Security Agency.

## CONFLICT OF INTEREST STATEMENT

CT has received funding from ViiV Healthcare via Penta Foundation for projects. CAS has received funding for membership of data safety and monitoring boards, advisory boards, and for preparation of educational materials from Gilead Sciences, ViiV Healthcare, and Janssen‐Cilag. All other authors declare no competing interests.
